# Pharmacological evidences for the stimulation of calcium-sensing receptors by nifedipine in gingival fibroblasts

**DOI:** 10.4103/0976-500X.77111

**Published:** 2011

**Authors:** Toshimi Hattori, Toshiaki Ara, Yoshiaki Fujinami

**Affiliations:** *Department of Dental Pharmacology, Matsumoto Dental University, Shiojiri 399-0781, Japan*

**Keywords:** Calcium-sensing receptor, gingival fibroblast, nifedipine

## Abstract

**Objective:**

To investigate pharmacologically whether CaSRs are involved in the Ca^2+^ antagonist-induced [Ca^2+^]i elevation in gingival fibroblasts.

**Materials and Methods:**

Gin-1 cells, normal human gingival fibroblasts, were used as the material. The [Ca^2+^] i was measured with fura-2/AM, a Ca^2+^-sensitive fluorescent dye.

**Results:**

At first, we confirmed the existence of CaSRs in these cells by showing that [Ca^2+^] i was elevated by high concentrations of extracellular Ca^2+^ and by prototypic agonists of the CaSR such as gentamicin. The action of gentamicin was antagonized by inhibitors of phospholipase C (PLC), inositol trisphosphate (IP_3_) receptors, NSCCs, and, importantly, by the CaSR antagonist, NPS2390. Furthermore, the action of gentamicin was potentiated by activators of PLC and protein kinase C (PKC). This confirmed the pathway components mediating Ca^2+^ responses to a known agonist of the CaSR. We then investigated whether nifedipine (an L-type Ca^2+^ channel blocker) stimulates CaSRs to elevate [Ca^2+^] i via a similar mechanism. Nifedipine Ca^2+^ responses were dose-dependently blocked by NPS2390 and by the same inhibitors of PLC, IP_3_ receptors, and NSCCs that disrupted the action of gentamicin. Calphostin C (a PKC inhibitor) and TMB-8 (an inhibitor of Ca^2+^ release from stores) also inhibited the nifedipine-induced [Ca^2+^] i elevation.

**Conclusion:**

These findings suggest that CaSRs are involved in the nifedipine-induced [Ca^2+^] i elevation in gingival fibroblasts.

## INTRODUCTION

Gingival overgrowth is known to be a common side effect of calcium (Ca^2+^) channel antagonists.[1] This effect is closely connected with the proliferation of gingival fibroblasts, in which Ca^2+^ antagonists elevate the intracellular Ca^2+^ concentration ([Ca^2+^]i). Inhibitors of nonselective cation channels [NSCCs; e.g., SKF-96365, mercury chloride, and flufenamic acid) significantly reduce the [Ca^2+^]i increase caused by isradipine, a dihydropyridine-derived Ca^2+^ channel antagonist,[2] as do U73122 (an inhibitor of phospholipase C (PLC)][3] and xestospongin C (an antagonist of the inositol trisphosphate (IP_3_) receptor.[4] This suggests that Ca^2+^ channel antagonists like isradipine may elevate [Ca^2+^]i in gingival fibroblasts via the activation of NSCCs and mobilization of Ca^2+^ from intracellular stores.[5]

Ca^2+^ channel antagonists such as nifedipine (a structural analog of isradipine) have been shown to inhibit the secretion of parathyroid hormone (PTH) and thus to affect serum PTH levels.[6] This may be linked to the observation that nifedipine also increases [Ca^2+^]i in parathyroid adenoma cells.[7] A key mediator of parathyroid Ca^2+^ signaling is the calcium-sensing receptor (CaSR), which stimulates the influx of Ca^2+^ through NSCCs and the release of Ca^2+^ from intracellular stores to elevate [Ca^2+^]i.[8] This mechanism of action bears a striking similarity to that employed by isradipine in gingival fibroblasts.[5]

In addition to the parathyroid gland,[9] CaSRs are also found in periodontal cells such as gingival keratinocytes,[10] osteoblasts,[11] and osteoclasts.[12] Given the similarity between the Ca^2+^ signaling pathways downstream of isradipine (in gingival fibroblasts) and the CaSR (in the parathyroid gland), we postulated that CaSRs may be involved in Ca^2+^ channel blocker-activated Ca^2+^ signaling in gingival cells. We show here that nifedipine, the archetypal dihydropyridine-derivative calcium channel inhibitor, indeed acts via a CaSR pathway to elevate [Ca^2+^]i in gingival fibroblasts.

## MATERIALS AND METHODS

### Chemicals

Tissue culture reagents were purchased from Gibco BRL (Rockville, MD, USA) and fura-2/AM and EGTA were obtained from Dojindo Laboratories (Kumamoto, Japan). Nifedipine, gentamicin, neomycin, spermine, lanthanum chloride, verapamil hydrochloride, phorbol 12-myristate 13-acetate (PMA), and quinoxaline-2-carboxylic acid adamantan-1-ylamide (NPS2390) were purchased from Sigma (St. Louis, MO, USA). 1-[β-(3-(4-methoxyphenyl)propoxy)-4-methoxyphenethyl]-1H-imidazole hydrochloride (SKF-96365), *n* -(3-trifluoromethylphenyl)-2, 4, 6-trimethylbenzenesulfonamide (*m*-3M3FBS), and 1-[6-[((17β)-3-methoxyestra-1, 3, 5 [10] -trien-17-yl)amino]hexyl]-1H-pyrrole-2,5-dione (U-73122) were obtained from Calbiochem (CA, USA). All other chemicals were supplied by Nacalai Tesque (Kyoto, Japan). These chemicals were dissolved in dimethyl sulfoxide (Sigma) as stock solutions, and thereafter, added in the perfusate.

### Cell culture

Normal human gingival fibroblast Gin-1 cells were obtained from Dainippon Pharmaceutical Co. Ltd. (Osaka, Japan). The cells were cultured for 3–6 days in Dulbecco’s modified Eagle medium (Medium 41, Dainippon Pharmaceutical Co. Ltd.). The cells (5 × 10^3^ per cm^2^) were plated on fibronectin-coated glass cover slips adhered to a flexiperm disc (Greiner Bio-One GmbH, Göttingen, Germany). The medium was supplemented with 10% fetal bovine serum in a humidified atmosphere of 95% air and 5% CO_2_ at 37°C. The medium also contained antibiotics (50 U/ml penicillin and 50 µg/ml streptomycin; Sigma), and was changed at intervals of 2–3 days.

### [Ca^2+^]i measurement

[Ca^2+^]i was measured with the Ca^2+^-sensitive fluorescent dye, fura-2/AM (Dojindo Laboratories). The cells were kept in a buffer consisting of 135 mM NaCl, 5 mM KCl, 1 mM CaCl_2_, 1 mM MgCl_2_, 10 mM glucose, and 20 mM HEPES–NaOH (pH 7.4), and loaded with the dye by incubation in 5 µM fura-2/AM for 45 min at 37°C. Cells were then washed to remove excess fura-2/AM and then incubated in a fresh buffer (without fura-2/AM) for 15 min postincubation to allow intracellular cleavage of the acetoxymethylester conjugate (and thus activation) of fura-2. Excitation light from a xenon lamp was passed through a 340- or 360-nm filter. The emission wavelength for analysis was 510 nm. Changes in the fluorescence intensity of fura-2 in the cells were recorded with a video-imaging analysis system (FC-400, Furusawa Lab Appliance, Kawagoe, Japan). [Ca^2+^]i was determined as the ratio of the fluorescence stimulated by excitation at 340 nm/360 nm, compared with a standard calibration curve obtained using Calcium Calibration Buffer Kit I (Molecular Probes, Inc. Eugene, OR, USA).

To minimize the leakage of fura-2, cells were kept at 32°C during fluorescence measurement using a bath temperature controller (DTC-100A, DIA Medical System, Kunitachi, Japan). The cells were soaked in a flexiperm chamber containing 0.5 ml of saline and were perfused at 8.0 ml/min with a tubing pump system (Master flex 7524-10, Cole-Parmer Instrument Company, Barrington, IL, USA). Drugs were added to the perfusate at appropriate concentrations. The time of treatment with CaSR agonists was 1 min. To ensure that fura-2 fluorescence was maintained within the linear range (i.e., did not become saturated), we only selected analysis cells with the basal [Ca^2+^]i in the range of 50–200 nM.

### Statistical analyses

Data are represented as the mean value ± standard error of the mean (SEM) and the number of observations (*N*). Statistical analyses of the data were performed by Student’s two-sided paired *t*-test. Differences between the mean values were considered significant if the probability of error (*P*) was less than 0.05.

## RESULTS

### Existence of functional CaSRs in gingival fibroblasts

**Figure 1:**
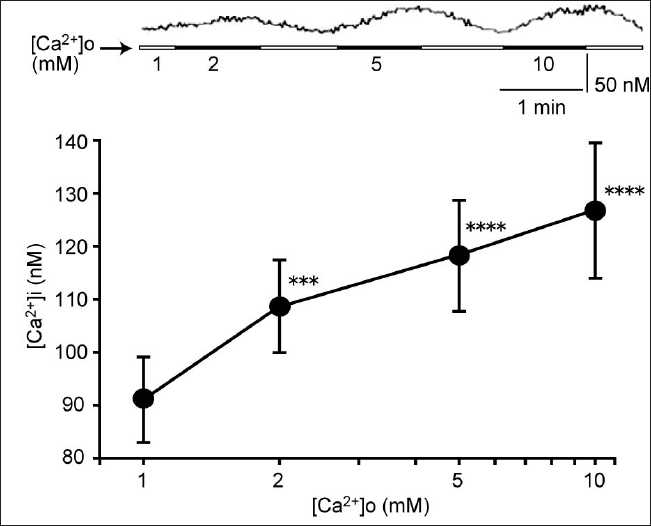
Effects of the high extracellular Ca^2+^ concentration ([Ca^2+^]o) on the [Ca^2+^]i of gingival fibroblasts. Application of Ca^2+^ (2–10 mM) in the perfusion buffer concentration dependently elevated the [Ca^2+^]i. The upper trace shows a time course of the [Ca^2+^]i. Data are mean ± SEM, N = 20. ***P < 0.005, ****P < 0.001 compare to corresponding pretreated values

Though the study was initially planned to carry out the CaSRs expressed in the Gin-1 cells used in this experiment, later the protocol was modified to evaluate [Ca^2+^]i changes following the administration of a range of concentrations (2–10 mM) of extracellular Ca^2+^. Figure 1 illustrates that extracellular Ca^2+^ dose-dependently elevates [Ca^2+^]i in these cells.

### Responsiveness of CaSRs to known agonists

**Figure 2:**
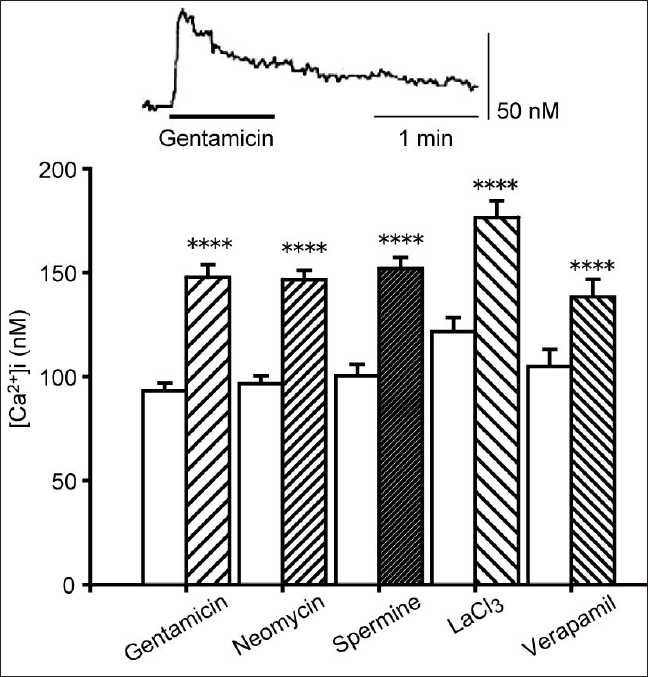
Elevation of the [Ca^2+^]i by calcium-sensing receptor agonists. Intracellular Ca^2+^ measurements were made in the presence (hatched bars) or absence (clear bars) of various compounds: gentamicin (500 µM), neomycin (300 µM), spermine (3 mM), LaCl_3_ (10 µM), and verapamil (25 µM). The upper trace shows a representative time course of the [Ca^2+^]i in the case of gentamicin application. Data are mean ± SEM, N = 32 (gentamicin, neomycin, spermine), 25 (LaCl_3_), or 20 (verapamil). ****P < 0.001compare to corresponding pretreated values

As shown in Figure 2, the CaSR agonists gentamicin (500 µM), neomycin (300 µM), spermine (3 mM), lanthanum chloride (LaCl_3_; 10 µM), and verapamil (25 µM) all significantly raised [Ca^2+^]i. We further tested the concentration-dependence of the response to verapamil. Increasing concentrations of verapamil (1, 5, and 25 µM) induced progressively larger [Ca^2+^]i elevations above basal (4.20 ± 1.01 nM; 9.65 ± 1.04 nM; and 33.6 ± 3.30 nM, respectively; *P* < 0.001 for all concentrations; *N* = 20 in each case). This demonstrates that known CaSR agonists mediate intracellular Ca^2+^ signaling in normal human gingival fibroblast Gin-1 cells.

### Delineating the pathway(s) of [Ca^2+^]i elevation by the CaSR agonist, gentamicin

**Figure 3:**
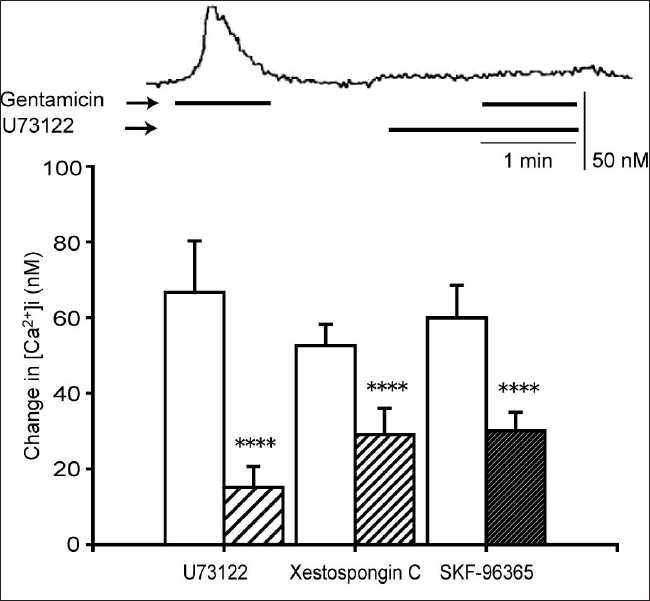
Effects of U73122, xestospongin C, and SKF-96365 on the gentamicin-induced [Ca^2+^]i elevation. Gentamicin (500 µM) was antagonized by U73122 (10 µM), xestospongin C (2 µM), and SKF-96365 (10 µM). The upper trace shows the effect of U73122 on the gentamicin-induced [Ca^2+^]i elevation. In the lower graph, white columns represent the [Ca^2+^]i elevated by gentamicin alone. Data are mean ± SEM, N = 8 (U73122), 14 (xestospongin C), or 32 (SKF-96365). ****P < 0.001 compare to corresponding pretreated values

The stimulation of CaSRs induces [Ca^2+^]i changes by promoting both the release of Ca^2+^ from stores and influx through NSCCs. We investigated the effects of inhibitors of these pathways on the [Ca^2+^]i changes elicited by the CaSR agonist, gentamicin. We used the PLC inhibitor, U73122 (10 µM);[3] the IP_3_ receptor antagonist, xestospongin C (2 µM);[4] and the NSCC blocker, SKF-96365 (10 µM).[13] As shown in Figure 3, each of these treatments strongly reduced the Ca^2+^ response to gentamicin.

**Figure 4:**
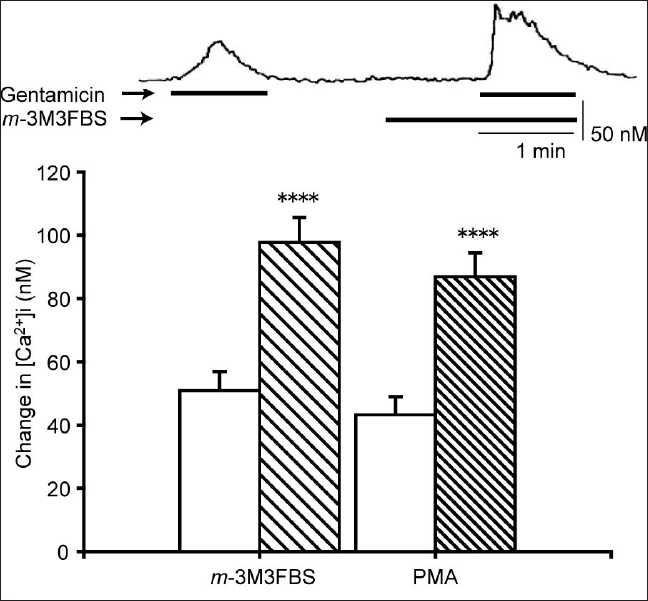
Enhancement of the gentamicin-induced [Ca^2+^]i elevation by m-3M3FBS and PMA. Gin-1 cells were stimulated with either gentamicin (500 µM) or gentamicin in combination with m-3M3FBS (20 µM) or PMA (20 µM). Upper panel: representative trace showing the effect of m-3M3FBS on the gentamicin-induced [Ca^2+^]i. Lower panel: effect of activators on the gentamicin-stimulated [Ca^2+^]i elevation. White columns represent the [Ca^2+^]i elevation stimulated by gentamicin alone; hatched bars denote gentamicin in combination with the activators. Data are mean ± SEM, N = 32 (m-3M3FBS) or 22 (PMA). ****P < 0.001 compare to corresponding pretreated values

It is known that the pathways mediating CaSR-mediated Ca^2+^ release from stores and influx through NSCCs involve PLC and protein kinase C (PKC), respectively. In addition to our findings with U73122 [Figure 3], the effects of *m*-3M3FBS (a PLC activator,[14] 20 µM) and phorbol myristate acetate (PMA, a PKC activator[15]) on gentamicin-induced Ca^2+^ responses were evaluated. Neither of these activators stimulated [Ca^2+^]i elevation alone but the co-administration of either *m*-3M3FBS or PMA together with gentamicin led to an approximately 100% enhancement of [Ca^2+^]i elevation compared with gentamicin alone [Figure 4].

**Figure 5:**
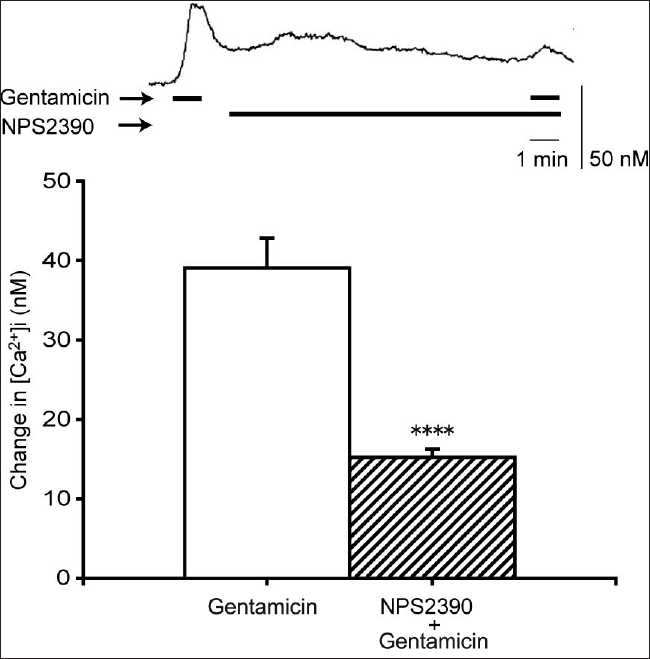
Effects of NPS2390 on the gentamicin-induced [Ca^2+^]i elevation. Gin-1 cells were pretreated with NPS2390 (10 µM) for 10 min prior to the application of gentamicin (500 µM). Upper panel: representative trace shows the effect of gentamicin on [Ca^2+^]i in the absence or presence of NPS2390. Lower panel: effect of NPS2390 on the gentamicin-stimulated Ca^2+^ elevation. Data are mean ± SEM, N = 22. ****P < 0.001 compare to corresponding pretreated values

The effect of NPS2390, a CaSR antagonist, on the gentamicin-induced [Ca^2+^]i elevation in normal human gingival fibroblast Gin-1 cells was evaluated [Figure 5]. Pretreatment with NPS2390 (10 µM) for 10 min[16] showed significant inhibition of the gentamicin-induced [Ca^2+^]i elevation.

### Characterization of nifedipine-activated Ca^2+^ signaling pathways

**Figure 6:**
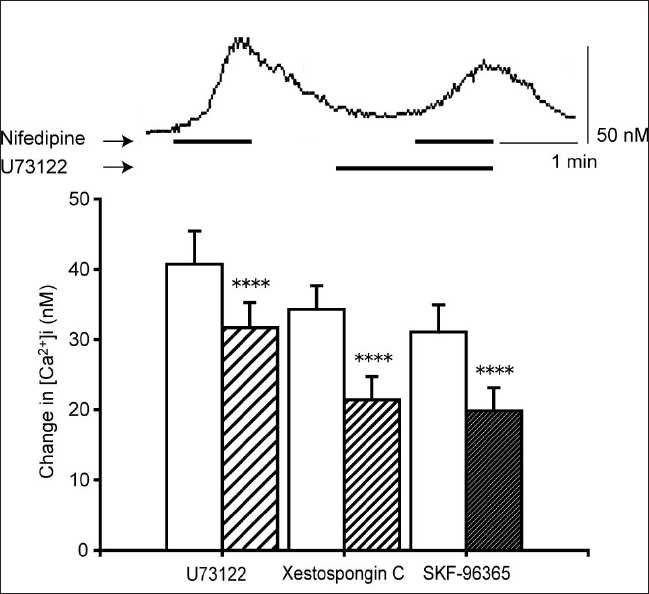
Inhibition of the nifedipine-induced [Ca^2+^]i elevation by pathway inhibitors. Cells were preincubated with U73122 (10 µM), xestospongin C (2 µM), or SKF-96365 (10 µM) prior to the application of nifedipine (10 µM). Upper panel: representative trace showing the effect of U73122 on the nifedipine-induced [Ca2+]i elevation. Lower panel: effect of nifedipine on [Ca^2+^]i in the absence (white bars) or presence (hatched bars) of inhibitors. Data are mean ± SEM, N = 12 (U73122), 16 (xestospongin C), or 20 (SKF-96365). ***P < 0.005, ****P < 0.001 compare to corresponding pretreated values

**Figure 7:**
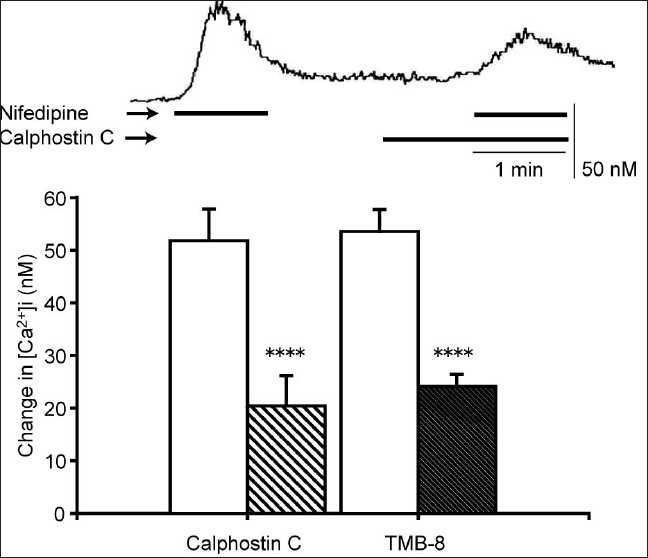
Inhibition of the nifedipine-induced [Ca^2+^]i elevation by calphostin C and TMB-8. Cells were incubated with calphostin C (2 µM) or TMB-8 (500 prior to the addition of nifedipine (10 µM). Upper panel: representative trace showing the effect of calphostin C on the nifedipine-induced [Ca^2+^]i elevation. Lower panel: Ca^2+^responses to nifedipine in the absence (white bars) or presence (hatched bars) of inhibitors. Data are mean ± SEM, N = 30 (calphostin C) or 42 (TMB- 8). ****P < 0.001 compare to corresponding pretreated values

**Figure 8:**
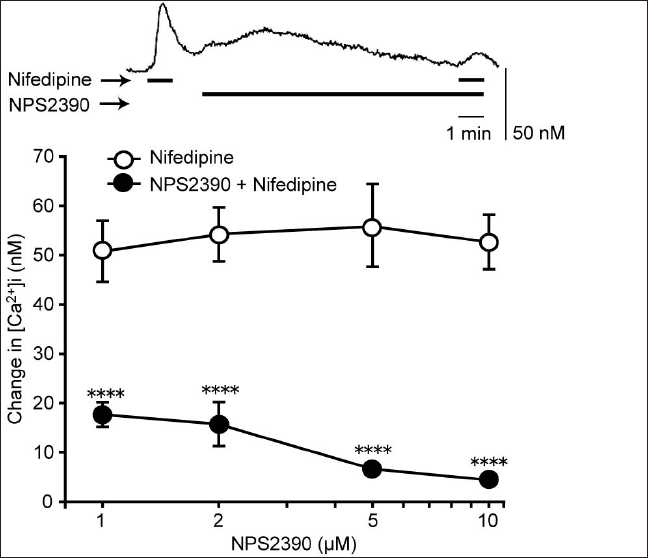
Antagonism of nifedipine responses by NPS2390. Cells were inhibited with various concentrations of NPS2390 (1–10 µM) prior to the addition of nifedipine. Upper panel: representative trace showing the inhibition of the nifedipine-induced [Ca^2+^]i elevation by NPS2390 (5 µM). Lower panel: [Ca^2+^]i elevation by NPS2390 (5 µM). Lower panel: Ca^2+^ responses to nifedipine in the absence (○) and presence (·) of NPS2390. Data are mean ± SEM, N = 29 (1 µM), 42 (2 26 (5 or 30 (10 ****P < 0.001 compare to corresponding pretreated values

Nifedipine (10 µM) also stimulated CaSRs, and U73122 (10 µM), xestospongin C (2 µM), and SKF-96365 (10 µM) significantly inhibited the [Ca^2+^]i elevation induced by 10 µM of nifedipine [Figure 6]. These effects were qualitatively the same as those for the gentamicin-induced [Ca^2+^]i elevation. A similar effect was observed with calphostin C (a PKC inhibitor)[17] and TMB-8 (a Ca^2+^ store release inhibitor).[18] Calphostin C (2 µM) and TMB-8 (500 µM) showed a significant inhibition of the nifedipine-induced [Ca^2+^]i elevation [Figure 7]. The cell culture which was incubated for 10 min with NPS2390 between two pulses of 10 mM nifedipine separated by 12 min [Figure 8, upper panel] also inhibited the nifedipine-induced [Ca^2+^]i elevation, in a concentration-dependent manner (concentration range: 1–10 µM; Figure 8, lower panel). It is important to note that, in a similar experiment where cells were perfused for 10 min with the vehicle instead of NPS2390, the sequential administration of two pulses of nifedipine (10 µM) separated by 12 min caused changes in the [Ca^2+^]i of 61.78 ± 10.99 nM and 58.71 ± 11.04 nM, respectively (*N* = 34, data not shown). There was no significant difference between the first and second responses, demonstrating that the effect of NPS2390 was not an artifact of, for example, the store depletion.

## DISCUSSION

In addition to their function in the parathyroid gland, CaSRs are expressed in several other tissues where they are involved in various cellular processes such as proliferation, differentiation, and hormone secretion.[8] The stimulation of CaSRs results in the modulation of several signaling pathways depending on the tissue in which they are expressed. Given that Ca^2+^ signaling pathways downstream of the CaSR bear a striking similarity to those reported for certain calcium channel blockers, it is possible that CaSR signaling pathways are involved in the nifedipine-induced proliferation of gingival fibroblasts. More precisely, we inferred that the nifedipine-induced [Ca^2+^]i elevation may be due to the direct stimulation of the CaSR by nifedipine. We conducted the present study to investigate this hypothesis.

Evidence for the presence of a CaSR can be obtained using functional biological assays, e.g., the demonstration that a high extracellular Ca^2+^ concentration causes a concomitant elevation of [Ca^2+^]i,[19] an effect we have also noted here in Gin-1 cells. Several CaSR agonists have been discovered such as gentamicin,[20] neomycin,[20] spermine,[21] LaCl3,[22] and verapamil,[18] all of which raise [Ca^2+^]i in the Gin-1 cells used in this study. Importantly, the CaSR antagonist, NPS2390,[23] inhibited the gentamicin and nifedipine responses. Together, these data confirm the presence of CaSRs in these cells.

Verapamil is an interesting member of this group of compounds in that it is a structural member of the phenylalkylamine family of compounds, which were an important intermediate in the development of calcimimetic drugs.[24] In addition, it is a Ca^2+^ channel antagonist, and evokes gingival overgrowth. Therefore, the concentration-dependent changes in [Ca^2+^]i in response to verapamil are further evidence of the fact that calcium channel blockers are able to activate CaSRs in gingival fibroblasts.

The agonist-induced activation of the CaSR causes [Ca^2+^]i increases that arise from two distinct mechanisms: (a) mobilization of Ca^2+^ from intracellular stores (e.g., endoplasmic reticulum) and (b) influx of extracellular Ca^2+^ through a Ca^2+^ -permeable NSCC.[19,25] The predominant pathway may vary depending on the tissue in which the receptor is expressed. Most CaSRs are linked to a G protein-mediated second messenger pathway, which activates PLC.[9] PLC hydrolyzes phosphatidylinositol 4,5-bisphosphate to IP_3_ and diacylglycero1 (DAG). IP_3_ mobilizes stored Ca^2+^ from the endoplasmic reticulum, resulting in an increase in [Ca^2+^]i, while PKC, under the control of DAG,[26] activates the NSCC.[27] From our results with U73122, xestospongin C, SKF-96365, *m*-3M3FBS, and PMA, we conclude that gentamicin-stimulated Ca^2+^ responses are mediated by pathways involving PLC, IP_3_ receptors, NSCCs, and PKC. The involvement of PLC and IP_3_ implicates intracellular stores as a source of calcium for these responses. Bae *et al*.[14] demonstrated that *m*-3M3FBS induces intracellular calcium increases in human neutrophils by stimulating the release of stored intracellular calcium. Similarly, Wonneberger *et al*.[19] have shown in vascular smooth muscles that a high extracellular Ca^2+^ concentration increases [Ca^2+^]i by activating CaSRs, leading to vasoconstriction. The blockade of this response by U73122 lends further support to the conclusion that the PLC–IP_3_ pathway plays a major role in signaling the downstream of the CaSR.

Next, we tested whether the nifedipine-induced [Ca^2+^]i elevation is due to CaSR stimulation. The most direct evidence for this was the efficacy of NPS2390 in inhibiting the nifedipine-induced [Ca^2+^]i elevation. NPS2390 has been used previously to confirm the presence of CaSRs, most recently by Cortijo *et al*.[16] who observed that NPS2390 abolished nickel-induced [Ca^2+^]i responses via CaSRs in A549 human airway epithelial cells. The nifedipine-induced [Ca^2+^]i elevation was also blocked by many of the same pathway inhibitors that disrupted signaling in response to gentamicin, a known agonist of the CaSR, suggesting a similar mechanism of action for these two agonists.

Here we present evidence that the CaSR is present in our Gin-1 cells and that nifedipine acts through it to mediate intracellular signaling events similar to those seen during gentamicin treatment. It may be necessary to perform molecular biological and immunohistochemical experiments to further elucidate the detailed relationship between nifedipine and CaSRs. However, our findings represent an important step in our understanding of how such calcium channel blockers induce fibroblast proliferation in the gingiva, and, consequently, gingival outgrowth.
